# Daptomycin Resistant *Staphylococcus aureus* Clinical Strain With Novel Non-synonymous Mutations in the *mprF* and *vraS* Genes: A New Insight Into Daptomycin Resistance

**DOI:** 10.3389/fmicb.2018.02705

**Published:** 2018-11-06

**Authors:** Artur J. Sabat, Marco Tinelli, Hajo Grundmann, Viktoria Akkerboom, Monica Monaco, Maria Del Grosso, Giulia Errico, Annalisa Pantosti, Alexander W. Friedrich

**Affiliations:** ^1^Department of Medical Microbiology, University Medical Center Groningen, University of Groningen, Groningen, Netherlands; ^2^Division of Infectious and Tropical Diseases, Hospital of Lodi, Lodi, Italy; ^3^Institute for Infection Prevention and Hospital Epidemiology, Medical Center – University of Freiburg, Faculty of Medicine, University of Freiburg, Freiburg, Germany; ^4^Department of Infectious Diseases, Istituto Superiore di Sanità, Rome, Italy

**Keywords:** whole-genome sequencing, *Staphylococcus aureus*, daptomycin, SNP analysis, MprF

## Abstract

**Objectives:** Daptomycin (DAP) resistance in *Staphylococcus aureus* is uncommon but there are increasing reports of the emergence of resistance during DAP therapy. Most clinical DAP-resistant *S. aureus* isolates investigated carried mutations in the *mprF* gene. The aim of this study was to identify mutations between a clinical pair of methicillin-susceptible *S. aureus* (MSSA) isolates (DAP-susceptible and DAP-resistant). Additionally, the activity of genes previously associated with DAP resistance was assessed.

**Materials and Methods:** Two MSSA isolates from patient with left-sided endocarditis were analyzed by whole genome sequencing (WGS) and reverse transcription-quantitative real-time PCR (RT-qPCR). The first isolate, DAP-susceptible, was obtained before initiation of treatment and the second isolate, DAP-resistant, was recovered after 4 weeks of DAP therapy.

**Results:** Comparison of complete genomes of DAP-susceptible and its DAP-resistant variant identified two non-synonymous and one synonymous mutations. The non-synonymous mutations consisted of a S829L substitution in *mprF* and a T331I substitution in *vraS*. The RT-qPCR experiments revealed an increased expression of *vraS, dltA, mprF*, and *sceD* genes in DAP-resistant variant. Strikingly, the expression of *dltA* and *mprF* genes was significantly downregulated by DAP.

**Conclusion:** The *mprF* and *vraS* genes were previously associated with DAP resistance, however, none of the mutations described in this study had been previously identified and linked to DAP resistance. Moreover, we provide a new insight into the DAP action on *S. aureus*, in which the expression of key genes in DAP resistance is decreased by the antibiotic.

## Introduction

Daptomycin (DAP) is an alternative to vancomycin for invasive methicillin-resistant *Staphylococcus aureus* (MRSA) infections or for serious methicillin-susceptible *S. aureus* (MSSA) infections in patients who are allergic to beta-lactams. The DAP non-susceptibility in *S. aureus* (referred to as DAP resistance in this study for the ease of presentation) is an increasing problem and several reports have described the emergence of resistance during DAP therapy (Lee et al., 2010; Mammina et al., 2010). The current knowledge suggests that DAP resistance in *S. aureus* is complex and results from mutational changes in a number of different genes. Most clinical DAP-resistant *S. aureus* isolates (MICs of >1 μg/ml) investigated to date, harbored mutations in *mprF*, typically in the form of single-nucleotide polymorphisms (SNPs) (Jones et al., 2008; Ernst et al., 2009; Mishra and Bayer, 2013). The *mprF* gene encodes a bifunctional membrane protein that catalyzes the synthesis and translocation (flipping) of the positively charged phospholipid lysyl-phosphatidylglycerol within its cell membrane. The amino acid substitutions in the MprF protein identified in the strains showing resistance to DAP lead to altered cell membrane phospholipid profiles. It results in a cell membrane positive charge increase and changes in cell membrane fluidity (Mishra et al., 2009). The *dltABCD* operon is involved in the addition of D-alanine to teichoic acids in many Gram-positive bacteria (Ernst et al., 2009). Mutations in the *dlt* operon and/or altered expression of its genes lead to a cell surface positive charge increase, as in the case of the *mprF* mutations. Data from numerous studies have suggested that charge repulsion arising from the *mprF*- and *dlt*-mediated enhancement of positive surface charge is mainly responsible for DAP-resistant phenotype among *S. aureus* strains (Jones et al., 2008; Patel et al., 2011; Peleg et al., 2012; Gasch et al., 2013; Yang et al., 2013; Cafiso et al., 2014; Kang et al., 2017; Ma et al., 2018). It has recently been discovered that deletion of the *clpX* gene caused a small reduction in DAP susceptibility (Bæk et al., 2014). The highly conserved ClpX chaperone facilitates protein folding and with the ClpP protease, forming the ClpXP protease, controls cell size and is required for growth of *S. aureus* at low temperature (Stahlhut et al., 2017). Other determinants involved in DAP resistance include genes that encode enzymes associated with phospholipid metabolism, such as phosphatidylglycerol and cardiolipin synthetases (*pgsA* and *cls*, respectively) (Fischer et al., 2011). There are also two-component regulatory systems like WalKR also known as YycFG, VraSR or GraRS that directly or indirectly modulate transcription of a number of genes encoding proteins involved in wall metabolism and permeability (Tran et al., 2015). The emergence of mutations in these regulatory genes have been also associated with DAP resistance in *S. aureus* (Friedman et al., 2006; Mehta et al., 2012).

The main purpose of this study was to apply whole genome sequencing (WGS) to a clinical pair of MSSA isolates (DAP-susceptible and DAP-resistant) to detect genome-wide DNA sequence polymorphisms associated with DAP resistance. Additionally, the gene expression profiles of determinants previously linked with DAP resistance were investigated. Furthermore, the net cell-surface charge as the main mechanism responsible for the DAP-resistant phenotype in MSSA was assessed.

## Materials and Methods

### Isolates

The isolates were obtained from a 50-year-old male, with a history of alcohol abuse and several comorbidities, who had a mitral valve replacement and in the following 6 months experienced two episodes of left-sided endocarditis, diagnosed according to current guidelines (Habib et al., 2009). In the last episode, *Enterococcus faecalis* and MSSA (isolate IT1-S) were obtained from blood cultures. As the patient had previously shown an allergic reaction to penicillins, treatment was instituted with gentamycin (discontinued after 2 weeks) and DAP at the dose of 500 mg daily. After 4 weeks the patient’s conditions remained serious, with no improvement. Blood culture yield an MSSA (isolate IT4-R) that was resistant to DAP (Table 1).

**Table 1:** Characteristics of the study isolates.

*S. aureus* isolate	Isolation	Source	Infection	MIC μg/ml (broth microdilution method) of selected antibiotics			*spa* type	MLST
						
				Vancomycin	Daptomycin	Clindamycin		
IT1-S	30-04-2013	Blood	Endocarditis	1	0.5	0.5	t223	ST22
IT4-R	01-06-2013	Blood	Endocarditis	1	2	0.25	t223	ST22


### Antimicrobial Susceptibility Testing

Antimicrobial susceptibility was tested by the broth microdilution method using the customized microplates ITGPOSF2 (Biomedical Service s.r.l., Scorzé, Venice, Italy) according to the manufacturer’s instructions. The antibiotics tested were ampicillin, ampicillin/sulbactam, cefoxitin, ceftaroline, clindamycin, DAP, erythromycin, fusidic acid, gentamicin, levofloxacin, mupirocin, oxacillin, rifampicin, tigecycline, trimethoprim/sulfamethoxazole and vancomycin. The breakpoints indicated by the EUCAST guidelines were applied^1^.

### Next-Generation Sequencing (NGS)

Next-generation sequencing (NGS) was carried out on the Illumina MiSeq system using 300 × 2 paired-end reads. Sequence libraries were created using the standard Illumina Nextera XT library creation kit. The SeqMan NGen software version 11.2.1 (DNASTAR) was used for *de novo* assembly of the reads and the resulting contigs were ordered by Mauve Contig Mover. The remaining gaps between contigs were closed by PCR amplification and Sanger sequencing allowing for the analysis of fully closed chromosomes and plasmids. Manual sequence editing was conducted using the SeqBuilder software (DNASTAR). The DNA sequences were aligned using the MegAlign (DNASTAR) and BLASTn software.

### *spa* Typing and MLST

The procedure was conducted as previously described (Aires-de-Sousa et al., 2006). The *spa* types were assigned using Ridom StaphType software version 1.4.6 (Ridom GmbH, Würzburg, Germany). The MLST STs were assigned through the publicly available MLST server^2^ on the basis of WGS data.

### RNA Sample Collection and Gene Expression Analysis Using Reverse Transcription and Quantitative Real-Time PCR (RT-qPCR)

Each isolate was grown in triplicate in Mueller Hinton Broth (MHB) in the absence or presence of DAP. The overnight cultures were diluted to produce an inoculum concentration of approximately 10^8^ cfu/ml. The inoculum was then diluted 1:100 in MHB supplemented with 50 μg/ml CaCl_2_ or in MHB supplemented with 0.08 μg/ml DAP and 50 μg/ml CaCl_2_. It resulted in starting number of the cells approximately 10^6^ cfu/ml in each culture. Cells were then harvested from the IT1-S and IT4-R cultures grown to mid exponential (∼10^8^ cfu/ml, after 5.5 h and 4 h, respectively), late exponential (∼10^9^ cfu/ml, after 8 h and 6 h, respectively) and stationary (∼10^10^ cfu/ml, after 14 h and 12 h, respectively) phase. The cell concentration was confirmed by colony counting after plating and incubation onto blood agar plates. Total RNA was isolated using the RNeasy Mini kit (Qiagen) according to the manufacturer’s instructions. RNA was quantified using Qubit (Thermo Fisher Scientific) and the quality of the RNA extracted was assessed by TapeStation 2200 (Agilent Technologies). The Agilent TapeStation 2200 system, which is an automated instrument for nucleic acid gel electrophoresis, assigns RNA Integrity Number (RIN) values ranging from 1 to 10, with 10 being the highest quality. Only samples with preserved 16S and 23S peaks and RIN values >8 were selected for gene expression analyses. The RIN values >8 indicate intact, high quality RNA samples for downstream applications (Fleige and Pfaffl, 2006). Total RNA was further treated with Baseline-ZERO DNase (Epicentre) followed by the RNeasy MinElute Cleanup kit (Qiagen) according to the manufacturer’s instructions. The absence of contaminating DNA was verified by PCR. For RT-qPCR analyses, an iTaq Universal SYBR Green One-Step Kit (Bio-Rad Laboratories) was used. Each reaction mix with a volume of 20 μl was prepared with 300 nM each primer (final concentration) and 20 ng of template RNA. A CFX96 Touch Real-Time PCR detection system (Bio-Rad Laboratories) was used for the measurements using a protocol with the following thermal cycling conditions: reverse transcription reaction at 50°C for 10 min and polymerase activation and DNA denaturation at 95°C for 1 min, followed by 35 cycles of denaturation at 95°C for 10 s and annealing/extension at 60°C for 30 s. After the last amplification cycle, a melting curve analysis was carried out by heating from 65 to 95°C in increments of 0.5°C/s. Negative controls (without template or reverse transcriptase enzyme) were included in each run. Gene-specific primers (Table 2) for the genes used in RT-qPCR experiments were designed using SeqBuilder software (version 15.0.1) from DNASTAR. Calculations of primer efficiencies were performed using the software CFX Manager version 3.1 (Bio-Rad Laboratories). Fold changes in the expression levels of the investigated genes were normalized in relation to the levels of *gyrB* mRNA. The relative changes in gene expression were quantified using the Pfaffl method (Pfaffl, 2001): gene expression ratio = (*E*_target_)^ΔCt^ ^target(control^^-^^sample)^/(*E*_reference_)^ΔCt^ ^reference(control^^-^^sample)^, where *E*_target_ is the amplification efficiency of target (gene of interest), *E*_reference_ is the amplification efficiency of reference (*gyrB*), Ct is the point at which the fluorescence rises above the background fluorescence, ΔCt target is the Ct deviation of the control minus the sample of the target gene transcript, and ΔCt reference is the Ct deviation of the control minus the sample of the reference gene transcript. The statistical significance of differences in the results was analyzed with the Student *t*-test and *P*-values of ≤0.05 were considered significant.

**Table 2:** Primers used in the RT-qPCR study.

Gene description (designation)	Gene position in the chromosome	Primer sequence	Primer position in the gene
DNA gyrase subunit B (*gyrB*)	5034–6968	F: TGAAGCATTAGCTGGTTATG	5204–5223
		R: ACGTTTTCTTCATCACGTTC	5679–5698
Two-component sensor histidine kinase (*vraS*)	1894439–1895482 (complement)	F: TGGTTCAATGCTCATCTTAG	1895440–1895459
		R: CTTTGATAGCAGATAGCATC	1894960–1894979
Bifunctional lysyl-phosphatidylglycerol flippase/synthetase (*mprF*)	1322308–1324830	F: ATCAACCGTATGTCCCTTG	1322449–1322467
		R: ATGAGCGTCAACAATTACAC	1322960–1322979
Transglycosylase (*sceD*)	2103087–2103782 (complement)	F: GCAGTAGGTTTAGGAATCG	2103734–2103752
		R: GATGTTGGATTTACAGCATG	2103250–2103269
D-Alanine–poly(phosphoribitol) ligase subunit 1 (*dltA*)	844667–846124	F: GATGATAGGTGCCATTAAAG	844858– 844877
		R: CAAATGTTAATCGGTGTTGC	845339–845358
Cell wall metabolism sensor histidine kinase (*walK*)	25652–27478	F: GAGGTAACTATACGCAACG	26316–26334
		R: GGTGTACGTAACTCATGTG	26802–26820
DNA-binding response regulator (*graR*)	663682–664356	F: TGGGATTTTAATGTTGCTGG	663748–663767
		R: ATCACTAACAAATGCTTCATC	664228–664248
Cardiolipin synthase (*cls*)	1273147–1274628	F: TGTTAATGGATCAAGATGGC	1273499–1273518
		R: TCTAAAATAAATCGCAACTGC	1273983–1274003
CDP-diacylglycerol–glycerol-3-phosphate 3-phosphatidyltransferase (*pgsA*)	1227992–1228570	F: TTAGAGTAGTGTTAATACCAG	1228020–1228040
		R: TATTCAATACCAGATAAGATAG	1228512–1228533
ATP-dependent Clp protease ATP-binding subunit (*clpX*)	1658527–1659789 (complement)	F: GCGATTACAGAATTACCTAC	1659602–1659621
		R: TTCTTGGTTTGGATGTTTGC	1659094–1659113


### Relative Positive Surface Charge Determination

The cells were grown overnight (16 h) in Tryptic Soy Broth (TSB) medium in the absence of DAP, washed twice with 20 mM morpholinepropanesulfonic acid (MOPS) buffer (pH 7.0), and resuspended in the same buffer at an optical density at 600 nm (OD_600_) of 1.0. The cells were incubated with cytochrome c (Sigma) dissolved in 20 mM MOPS buffer (pH 7.0) using three different concentrations: 50 μg/ml, 100 μg/ml, and 500 μg/ml. Moreover, the cells incubated in 20 mM MOPS buffer (pH 7.0) without cytochrome c were used as a negative control. After 15 min of incubation, the cells were pelleted by centrifugation and absorbance of the supernatants was measured at 530 nm. The amount of remaining (unbound) cytochrome c in the supernatant was determined by comparison to a standard curve (serially diluted cytochrome c). Cytochrome c is a cationic peptide and there is a direct correlation between the amount of unbound cytochrome c detected in the supernatant and the positive charge of the bacterial surface (Peschel et al., 1999). The data were converted and shown as the percent amount of bound cytochrome c. Independent runs were performed in triplicate on separate days. Changes in cytochrome c binding were compared by the Student *t*-test. A *P*-value of ≤0.05 was considered significant. Statistical analysis was performed using the GraphPad software.

### Membrane Potential Assay

DAP-induced bacterial membrane depolarization was performed essentially as described previously (Silverman et al., 2003) by using the membrane potential-sensitive fluorescent dye 3,3-dipropylthiacarbocyanine [DiSC3(5); Thermo Fisher Scientific] with the following modifications. Briefly, isolates were grown at 37°C to early exponential phase (OD_600_, 0.2 to 0.3) in 50 ml of MHB. Cells were harvested by centrifugation, washed twice with 5 mM HEPES buffer (pH 7.2) supplemented with 50 μg/ml CaCl_2_ and resuspended in the same buffer (containing 50 μg/ml CaCl_2_) to an OD_600_ of 0.2. The dye DiSC3(5) was added to cell suspension to make a final concentration of 0.18 μM. The cells were incubated with the dye DiSC3(5) for 15 min at room temperature. Then KCl was added (100 mM final concentration) to equilibrate the cytoplasmic and external potassium ions (K^+^) concentrations. The 200-μl cell suspension aliquots were transferred to the wells of a white 96-well microplate. The desired DAP concentrations were subsequently added to the microplate wells and an increase in the fluorescence intensity at a wavelength of 670 nm was measured over 60 min using a Synergy HTX multimode microplate reader (BioTek). The fluorescence leakage (*F*_L_) was determined using equation as previously described by Cheng et al. (2014): *F*_L_ = (*F*_F_ -*F*_B_) - (*F*_I_ -*F*_B_), where *F*_F_ was the fluorescence intensity of the cell suspension over 60 min of treatment with DAP, *F*_I_ was the initial fluorescence intensity of the cell suspension with DAP, and *F*_B_ was the fluorescence intensity of the blank (only cells and the dye). Data were normalized relative to the maximum fluorescence leakage (expressed as 100%). Results were shown as the mean of triplicate measurements. Changes in membrane depolarization were compared by the Student *t*-test. A *P*-value of ≤0.05 was considered significant. Statistical analysis was performed using the GraphPad software.

### Nucleotide Sequence Accession Numbers

Chromosome and plasmid sequence data of the two *S*. aureus isolates were annotated using the NCBI Prokaryotic Genome Annotation Pipeline and deposited in GenBank^3^ under accession numbers CP028468–CP028471.

## Results

### Antibiotic Resistance

The first aim of our investigations was to determine the antibiotic susceptibility of the isolates recovered before and after treatment with DAP. The MIC values for selected antibiotics are presented in Table 1. Antibiotic susceptibility testing showed that isolate IT1-S (recovered before the DAP treatment) was susceptible to all the antibiotics tested with the exception of clindamycin that showed intermediate resistance (MIC = 0.5 μg/ml). MIC value for DAP was 0.5 μg/ml. Isolate IT4-R (recovered after the DAP treatment) was resistant to DAP (MIC = 2 μg/ml) and susceptible to all other antibiotics tested. Therefore, the mechanism conferring DAP resistance in IT4-R did not confer resistance to other antibiotics, including vancomycin, for which the two isolates showed the same MIC (Table 1).

### Molecular Characterization

This part of the study aimed at molecular typing of the isolates and comparing their virulence potential. The characteristics of the isolates are shown in Table 1. *spa* typing assigned *spa* type t223 to both isolates. From the WGS data, *in silico* MLST identified the isolates belonged to single sequence type (ST) 22. Moreover, the isolates were positive for toxic shock syndrome toxin-1, enterotoxin N, enterotoxin O and enterotoxin P. The full virulence profile of the IT1-S and IT4-R isolates is presented in Table 3. Both isolates showed exactly the same virulence potential as they carried the same set of the genes.

**Table 3:** Virulence profile of the IT1-S and IT4-R isolates.

Virulence factor	Position in chromosome	Protein function
**Adhesins**		
*spa*	75928–77478	Immunoglobulin G binding protein A
*sdrC*	560282–563191	Ser-Asp rich fibrinogen-binding protein C
*sdrE*	567737–571072	Ser-Asp rich fibrinogen-binding protein E
*vwb*	806446–807954	von Willebrand factor-binding protein
*eap*	900239–900673	Extracellular adherence protein
*atl*	976444–980217	Bifunctional autolysin Atl
*fib*	1082735–1083064	Fibrinogen-binding protein
*efb*	1087580–1087957	Extracellular fibrinogen-binding protein
*ebpS*	1477847–1479277	Cell surface elastin binding protein
*eap/map*	1951015–1953063	Extracellular adherence protein
*sdrH*	2025520–2026779	Ser-Asp rich fibrinogen-binding portein H
*eap*	2227772–2228197	Extracellular adherence protein
*sbi*	2437079–2438389	Immunoglobulin-binding protein
*fnbA*	2529493–2532540	Fibronectin-binding protein A
*clfB*	2677709–2680114	Clumping factor ClfB, fibrinogen binding protein
*cna*	2750354–2753344	Collagen adhesin precursor
**Toxins**		
*hla*	1090201–1091160	Alpha-hemolysin precursor
*SEntP*	1591838–1592542	Enterotoxin P
*SEntG*	1822884–1823660	Extracellular enterotoxin type G precursor
*SEntN*	1823943–1824698	Enterotoxin N
*SEntI*	1825661–1826389	Extracellular enterotoxin type I precursor
*SEntO*	1827424–1828188	Enterotoxin O
*tsst*	2010878–2011582	Toxic shock syndrome toxin-1
*hld*	2031459–2031593	Delta-hemolysin
*hlgA*	2438880–2439808	Gamma-hemolysin chain II precursor
*hlgC*	2440376–2441323	Gamma-hemolysin component C
*hlgB*	2441325–2442302	Gamma-hemolysin component B precursor
**Exoenzymes**		
*coa*	211498–212859	Staphylocoagulase precursor
*geh*	314970–317045	Glycerol ester hydrolase
*nuc*	810521–811207	Thermonuclease
*sspB*	970467–971648	Cysteine protease staphopain B
*sspA*	971730–972776	Serine V8 protease
*nucI*	1278542–1279075	Thermonuclease
*scpA*	1923362–1924528	Cysteine protease staphopain A
*scn*	1957164–1957377	Complement inhibitor SCIN
*sak*	1959588–1960079	Staphylokinase
*hysA*	2220411–2222837	Hyaluronate lyase
*lip*	2733778–2735820	Triacylglycerol lipase


### The *S. aureus* IT4-R Genome Carries Mutations That Can Be Linked to DAP Resistance

Subsequently, the genome sequences of the two isolates were compared to identify mutational changes responsible for the DAP resistance of the IT4-R isolate. The analyzed *S. aureus* isolates, IT1-S and IT4-R, had identical genomes in length, which included a 2,774,523-bp circular chromosome (32.8% G+C content) and a 34,104-bp circular plasmid (30.3% G+C content). The NCBI annotation pipeline revealed 2,895 genes in total in each genome. Whole-genome comparison of both isolates, DAP-susceptible and its DAP-resistant variant, identified three single nucleotide polymorphisms (SNPs), one in each of three genes. Two of the mutations were non-synonymous (leading to a change in the amino acid sequence in the translated gene product) while the third mutation was synonymous (without change in amino acid sequence). The non-synonymous mutations consisted of (i) a S829L substitution in *mprF* and (ii) a T331I substitution in *vraS*. The synonymous mutation was found in *sceD* (A→G at the nucleotide 105), the gene involved in cell wall turnover, growth and cell separation (Howden et al., 2010). Although *mprF* and *vraS* genes were previously associated with DAP resistance, none of the SNPs that we identified had been previously described and linked to DAP resistance.

### Comparison of Gene Expression Between the IT1-S and IT-4 Isolates

**FIGURE 1:**
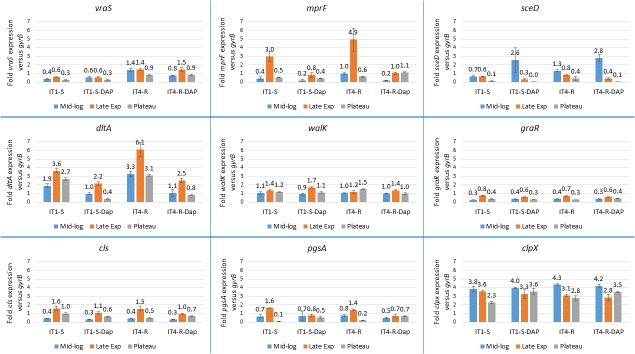
Expression analysis of genes previously linked with DAP resistance. Transcript levels of the analyzed genes were determined by RT-qPCR in relation to *gyrB* expression. Values represent the mean of results obtained for three replicates independently grown cultures for each isolate. Error bars indicate the standard deviation of comparisons between three replicates. IT1-S – isolate IT1-S grown in the absence of DAP, IT1-S-DAP – isolate IT1-S grown in the presence of 0.08 μg/ml daptomycin, IT4-R – isolate grown in the absence of daptomycin, IT4-R-DAP – isolate IT1-S grown in the presence of 0.08 μg/ml daptomycin. Mid-log, mid-exponential growth phase; Late Exp, late exponential growth phase; Plateau, stationary growth phase.

We wanted to know if the mutations, which arose in genomic DNA during DAP therapy had an influence on gene expression. The gene expression levels of IT4-R were compared to that of its parent strain IT1-S during mid-exponential, late exponential and stationary growth phases using RT-qPCR (Figure 1). All three genes, in which the SNPs were identified in this study (*mprF, sceD* and *vraS*) and the genes previously linked with DAP resistance (*dltA, walK, graR, cls, pgsA* and *clpX*) were investigated. The *vraS* gene showed significantly increased expression (P value ≤ 0.05) in IT4-R relative to IT1-S in all growth phases in both the absence and presence of DAP (Figure 1 and Supplementary Table S1). Likewise, *dltA, mprF* and *sceD* were upregulated in IT4-R but the differences between the two isolates were not always statistically significant (Supplementary Table S1). Other genes showed no difference in regulation between the two isolates. In conclusion, the increased expression of the *vraS, dltA, mprF*, and *sceD* genes was observed in the DAP-resistant variant compared to its parent DAP-susceptible strain.

### Influence of DAP on Gene Expression

It was shown previously that concentrations of some antibiotics below the MIC were able to modulate the expression of virulence-associated genes in *S. aureus* (Bernardo et al., 2004; Stevens et al., 2007; Turner and Sriskandan, 2015). However, DAP seemed to have no significant effects on virulence factor expression by *S. aureus* (Otto et al., 2013). In the current study, we wanted to explore if subinhibitory concentration of DAP could influence the expression of the genes linked with DAP resistance. Therefore, the expression levels of *mprF, sceD, vraS, dltA, walK, graR, cls, pgsA* and *clpX* were compared in IT1-S or in IT4-R in the absence and presence of DAP during different stages of growth (Figure 1). DAP significantly downregulated the expression of the *dltA* gene. With the exception of the stationary phase in IT4-R, the *mprF* gene was also downregulated in the presence of DAP. However, the difference was not significant for the mid-exponential phase in IT1-S (Supplementary Table S2). Based on these observations we could conclude that DAP downregulates the expression of *dltA* and *mprF* genes.

### The Effect of Growth Phase on Gene Expression

Throughout bacterial growth, the cell density, nutrient conditions, pH, and other factors are changing. Therefore, we analyzed the growth phase-dependent gene expression profiles (Figure 1). In both isolates in the absence or presence of DAP, the *sceD* gene showed the highest transcript level in the mid-exponential phase and during further growth its transcriptional activity was decreasing, while the highest and lowest transcriptional activity of *cls* was found in late exponential and mid-exponential growth phases, respectively (Figure 1). However, the differences between the growth phases were not always statistically significant (Supplementary Table S3). Moreover, *dltA* and *graR* were always the most active in the late exponential growth phase and *clpX* in the mid-exponential phase. In case of *dltA* and *graR* the differences were always statistically significant. In conclusion, independently on mutational changes during DAP therapy as well as presence or absence of DAP in medium, strong impact of the growth phase on gene expression was observed in case of *sceD* and *cls*.

### Relative Positive Surface Charge

**FIGURE 2:**
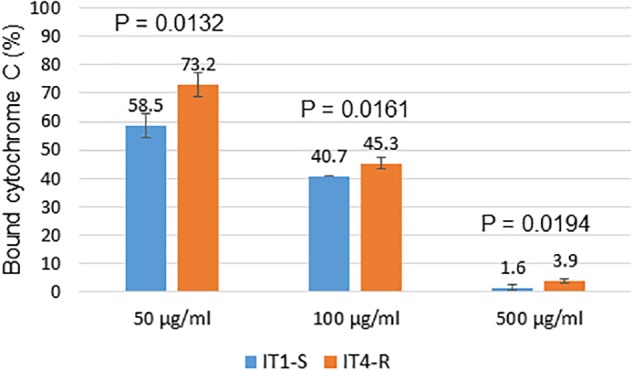
Binding analysis of cytochrome c to surface of *S. aureus* cells. The graph shows percentage of cytochrome c bound after incubation of *S. aureus* cells with different concentrations of cytochrome c. The data represent the means from three technical replicates. Error bars indicate the standard deviation of comparisons between three technical replicates. A *P*-value of ≤0.05 was considered significant.

The emergence of DAP-resistant *S. aureus* strains can occur by way of several different mechanisms involving the cell membrane and/or cell wall. Recent analysis of a panel of clinical DAP-resistant MSSA isolates showed that enhancement of positive surface charge, reducing DAP binding, may be the main mechanism of DAP resistance among the MSSA strains (Kang et al., 2017). To test this hypothesis the cytochrome c binding analysis was performed. Cytochrome c binding assay revealed that DAP-resistant isolate IT4-R had significantly decreased positive surface charge compared to its DAP-susceptible parental isolate IT1-S (Figure 2).

### DAP-Induced Membrane Depolarization

**FIGURE 3:**
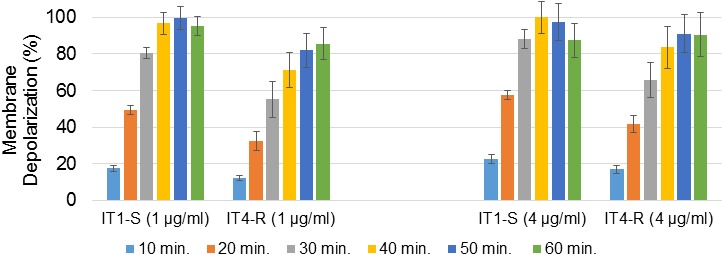
DAP-induced membrane depolarization. The graph shows percentage of membrane depolarization after 10, 20, 30, 40, 50, and 60 min of the antibiotic treatment. DAP concentrations in brackets. The data represent the means from three technical replicates. Error bars indicate the standard deviation of comparisons between three technical replicates.

The target of DAP is the bacterial cell membrane (Silverman et al., 2003). Once inside the membrane, DAP aggregates (Pogliano et al., 2012) leading to rapid depolarization and loss of membrane potential, and thus to bacterial cell death. We compared depolarization of the cell membrane of the IT1-S and IT4-R isolates to identify differences, which could explain higher resistance of the mutant to DAP. Changes in membrane depolarization were determined by membrane potential assay using a fluorescence dye, DiSC3(5). The fluorescence of DiSC3(5) decreases as it incorporates into polarized membranes because at high concentration the dye aggregates and self-quenches. With the addition of a membrane-disrupting agent, such as DAP, the dye is released from cells into the media, which in turn leads to fluorescence dequenching. DAP concentrations that were used in the membrane potential assay were 1 μg/ml and 4 μg/ml that corresponded to twice the DAP MIC for IT1-S and IT4-R, respectively. The addition of DAP into the assay medium gradually depolarized the membrane of the isolates (Figure 3). The rate of membrane depolarization was reduced in DAP-resistant isolate compared to that of DAP-susceptible isolate. However, statistically significant differences (*p* < 0.05) between IT1-S and IT4-R at the same time points were observed only between 10 and 40 min at the DAP concentration of 1 μg/ml, and between 10 and 30 min at the DAP concentration of 4 μg/ml. At the DAP concentration of 1 μg/ml, a maximum depolarization (100%) of the IT1-S membrane was observed after 50 min, while the antibiotic depolarized the membrane of IT4-R by 85.6% (compared to the maximum depolarization of IT1-S) after 60 min. At the DAP concentration of 4 μg/ml, the time to reach the maximum of depolarization was shorter for IT1-S (30 min), than for IT4-R (40 min). However, the peak of depolarization for IT4-R was lower than that of IT1-S (91.2% versus 100%). This assay demonstrated that both the total amount of membrane depolarization and the rate of depolarization were reduced in DAP-resistant isolate, which can explain higher resistance of the mutant to the antibiotic.

## Discussion

The MprF protein consists of an N-terminal transmembrane flippase domain, C-terminal catalytic synthase domain, and a central bifunctional domain bridging the flippase and synthase domains. The changes in amino acid sequence linked to DAP resistance of *S. aureus* were identified in all domains of MprF: G61V in the flippase domain (Peleg et al., 2012); S295L, P314L, S337L, T345A, and T345I in a central bifunctional domain (Friedman et al., 2006; Mehta et al., 2012; Peleg et al., 2012); and I420N and L826F in synthase domain (Peleg et al., 2012). Taking into account a frequency of above mutations they occur most often in a central bifunctional domain both in clinical and *in vitro* obtained isolates. Our study revealed a novel amino acid substitution in C-terminal catalytic synthase domain of the MprF protein at amino acid residue 829 (S829L), which was found in a clinical isolate with the DAP-resistant phenotype after prolonged treatment of a patient with endocarditis initially infected with a fully susceptible strain.

Although several molecular mechanisms of resistance to DAP have been proposed, mainly associated with phenotypic changes in cell wall and cell membrane, the basis of DAP resistance in *S. aureus* is still incompletely understood (Tran et al., 2015). This results from the fact that DAP resistance in *S. aureus* appears to be multi-factorial and strain specific. It has been proposed that charge repulsion of the DAP antibiotic molecule from the cell surface, which is associated with the increased positive charge of cell membrane and cell wall may be the main mechanism to explain DAP resistance in *S. aureus* strains (Jones et al., 2008; Patel et al., 2011; Peleg et al., 2012; Gasch et al., 2013; Yang et al., 2013; Cafiso et al., 2014; Kang et al., 2017; Ma et al., 2018). During the emergence of DAP resistance in *S. aureus*, the first SNPs in the genomic DNA appear most often in the *mprF* gene and are associated with a gain of enzymatic function, resulting in an increase in the positive charge of the cell membrane (Bayer et al., 2014). The mutant in this study displayed an amino acid substitution in the MprF protein and increased expression of the *mprF* gene. These two factors possibly reduced dissipation of membrane potential of the mutant in the presence of DAP, promoting resistance to the antibiotic molecules by electrostatic repulsion.

Another strategy utilized by *S. aureus* to enhance the positive surface charge is increased expression of the *dlt* operon (Bertsche et al., 2013; Cafiso et al., 2014; Mishra et al., 2014). The *dltABCD* gene products are involved in the incorporation of D-alanine into cell wall teichoic acids. Although in the RT-qPCR experiments we observed increased expression of the *dltA* gene in IT4-R, this isolate had higher capacity to bind cytochrome c than IT1-S. However, the test with cytochrome c measures the overall cell surface charge and not the membrane charge itself. Moreover, it has been previously shown that some DAP-resistant isolates with *mprF* mutations did not show significantly altered net surface charge (Bayer et al., 2015). It will be interesting to compare more clinical DAP-resistant *S. aureus* strains with non-increased surface charge relative to their respective isogenic susceptible strains by DAP-induced cell membrane potential assay to explore further the mechanisms of DAP resistance in *S. aureus*.

Second non-synonymous mutation identified in the genome of IT4-R was found in *vraS*, a gene of the two-component system VraSR, which positively modulates the regulation of cell-wall biosynthesis pathway in *S. aureus*. Moreover, the expression levels of *vraS* were significantly increased in IT4-R compared to that of DAP-susceptible counterpart. In a study recently published by Chen et al. (2018), the authors showed results indicating a causal relationship between the L431F substitution in the MprF protein and increased expression of the *vraSR* genes in the DAP-resistant strain leading concurrently to vancomycin resistance. But how the mutant MprF protein affected expression of *vraSR* and the cell wall-related genes was not elucidated. We can assume that in the IT4-R isolate the amino acid substitution in the MprF synthase domain (S829L) can lead not only to changes in the cell membrane of but also to pleiotropic effects, including upregulation of the VraSR system and the transglycosylase gene *sceD*, a feature involved in cell-wall turnover, resulting in increased resistance to DAP.

A previous study showed that the *mprF* sequence variations within the same clonal complex were not only associated with the DAP-resistant *S. aureus* strains but also with DAP-susceptible strains (Bayer et al., 2014). There is an increasing interest to use WGS for genotypic prediction of antimicrobial resistance, which has the potential to reduce bed-to-diagnosis time by eliminating culture-based antimicrobial susceptibility testing. However, accuracy of predictions depends on *a priori* knowledge of mutations in *mprF* as well as in other genes that are conferring the DAP-resistant phenotype in *S. aureus*. Our study identified two novel SNPs leading to the amino acid substitutions (S829L in MprF and T331I in VraS), which can be linked to *S. aureus* DAP resistance, improving our recognition of resistance signatures within core genomic determinants.

This study provided a new insight into the DAP action on *S. aureus* by way of decreasing the expression of *mprF* and *dltA*. The *dltABCD* operon, like the *mprF* gene, contributes to the staphylococcal net positive surface charge. It will be important to identify the mechanism by which DAP reduces transcriptional activity of the genes, which contribute to positive surface charge since our study showed that the antibiotic did not influence the expression of two-component systems, VraSR, GraSR, and WalKR, associated with resistance of *S. aureus* to DAP.

## Conclusion

We identified the new point mutation in the *mprF* gene leading to the amino acid substitution in the MprF protein that counteracts DAP antibiotic activity. Our results support the suggestion that *vraSR* contributes directly or indirectly to DAP resistance in *S. aureus*. Description of new mutations in the core genome, which can be linked with *S. aureus* resistance to DAP will allow development of better and more rapid diagnostic methods for identifying of drug resistance. The membrane potential assay demonstrated that both the total amount of membrane depolarization and the rate of depolarization were reduced in DAP-resistant isolate, possibly resulting in resistance to the antibiotic molecules by electrostatic repulsion. However, the reduced effect of DAP on membrane depolarization in DAP-resistant isolate, compared to its DAP-susceptible parent, was not associated with increased positive net cell surface charge, which warrants further investigation of a higher number of such isolates to better understand the mechanisms of DAP resistance in *S. aureus*. Finally, we shed a new light on the DAP action in *S. aureus*, in which the expression of key genes in DAP resistance is decreased by the antibiotic.

## Author Contributions

AS, MT, HG, AP, and AF designed the project. MT and AP provided the isolates, clinical and epidemiological data. VA, MM, MDG, and GE performed the experiments. AS wrote the manuscript. All authors interpreted the data and reviewed the manuscript.

## Conflict of Interest Statement

The authors declare that the research was conducted in the absence of any commercial or financial relationships that could be construed as a potential conflict of interest.

## Footnotes

1 http://www.eucast.org/

2 www.cbs.dtu.dk/services/MLST

3 www.ncbi.nlm.nih.gov/nucleotide
